# Association Between Hypertension, Dipping Status, and *ACE* and *AGTR1* Gene Polymorphisms in Adolescents with Type 1 Diabetes

**DOI:** 10.3390/biomedicines13030615

**Published:** 2025-03-03

**Authors:** Smiljka Kovacevic, Maja Jesic, Vera Zdravkovic, Stefan Djordjevic, Jelena Miolski, Vladimir Gasic, Marina Jelovac, Milena Ugrin, Sonja Pavlovic, Branko Subosic

**Affiliations:** 1 Endocrinology Department, University Children’s Hospital, 11000 Belgrade, Serbia; maja.jesic@udk.bg.ac.rs (M.J.); vera.zdravkovic@udk.bg.ac.rs (V.Z.); 2 Faculty of Medicine, University of Belgrade, 11000 Belgrade, Serbia; stf.djordjevic@gmail.com; 3 Rheumatology Department, University Children’s Hospital, 11000 Belgrade, Serbia; 4 Department of Pediatrics, General Hospital Stefan Visoki, 11420 Smederevska Palanka, Serbia; jelena.miolski@doctor.com; 5 Laboratory for Molecular Biomedicine, Institute of Molecular Genetics and Genetic Engineering, University of Belgrade, 11000 Belgrade, Serbia; vlada.gasic@imgge.bg.ac.rs (V.G.); marina.jelovac@imgge.bg.ac.rs (M.J.); milena.ugrin@imgge.bg.ac.rs (M.U.); sonja.pavlovic99@gmail.com (S.P.); 6 Biochemical Laboratory, University Children’s Hospital, 11000 Belgrade, Serbia; branko.subosic@udk.bg.ac.rs

**Keywords:** *ACE* gene polymorphism, *AGTR1* gene polymorphism, diabetes type 1, hypertension, adolescents

## Abstract

**Objectives**: This study aims to show the distribution of angiotensin-converting enzyme (*ACE*) rs1799752 (I>D) gene insertion/deletion (*I/D*) polymorphism and angiotensin II receptor type 1 (*AGTR1*) rs5186 (A>C) gene polymorphism in adolescents with hypertension (HT) and type 1 diabetes (T1D), as well as its association with hypertension and the diurnal variation of mean blood pressure (dipping phenomenon). **Methods**: A cross-sectional study was conducted involving 118 adolescents diagnosed with T1D who underwent clinical and laboratory investigations, genetic analyses, and 24 h ambulatory blood pressure monitoring. The genotype frequencies were compared between adolescents with HT and those with normal blood pressure. Additionally, the genotype frequencies were compared between dippers and non-dippers. **Results**: Patients with HT were more likely to be female and exhibited significantly poorer glycemic control and higher triglycerides, along with increased body mass index and daily insulin dosage. The prevalence of *ACE rs1799752* genotypes in the hypertensive group was 20% II, 66.7% ID, and 13.3% DD, which did not significantly differ from the normal blood pressure group with 29.1% II, 53.4% ID, and 17.5% DD (*p =* 0.625). The prevalence of *AGTR1 rs5186* genotypes in the hypertensive group was 53.3% AC, 40% AA, and 6.7% CC, which also did not significantly differ from the normal blood pressure group with 39.8% AC, 52.4% AA, and 7.8% CC (*p =* 0.608). A total of 46% of the patients exhibited non-dipping phenomena. The prevalence of non-dippers among the *ACE* genotypes was 13% DD, 33.3% II, and 53.7% ID (*p =* 0.369), while for the *AGTR1* genotypes, it was 50% AA, 42.6% AC, and 7.4% CC (*p =* 0.976). **Conclusions**: Our results indicate that in our adolescents with T1D, clinical and metabolic factors such as higher body mass index, triglycerides, suboptimal glycemic control, and female gender are more indicative of the development of hypertension than *ACE* and *AGTR1* gene polymorphisms. A potential reason for this finding could be the young age of the patients or the relatively small size of the study group. Future research involving larger sample sizes is needed to further investigate the genetic predisposition for the development of hypertension.

## 1. Introduction

Diabetes complications in children may appear before the clinical symptoms of type 1 diabetes (T1D) manifest but often remain clinically undetectable. This indicates that changes within the kidney, such as glomerular basal membrane thickening or oxidative damage to the vascular endothelium, which result in endothelial and vascular dysfunction and inflammation [1,2], can be detected by histopathological examination even prior to the appearance of clinical symptoms [3,4].

A higher prevalence of cardiovascular disease (CVD) among patients with T1D leads to higher mortality [5]. The prevalence of arterial hypertension in children and adolescents is estimated to range from 2% and 4% [6], with even higher rates reported in children with T1D [3,4]. Specifically, the prevalence of hypertension in children with T1D is estimated to be between 6% and 16% [7,8,9,10,11].

Hypertension is associated with diabetic nephropathy (DN) and left ventricular hypertrophy (LVH) in children and adults [12,13,14]. The loss of a nocturnal dip in blood pressure might influence the progression of hypertension and lead to organ damage in T1D.

Genetic predisposition and environmental factors contribute to the development of hypertension, particularly regarding the younger age at diagnosis, its severity, and longstanding complications [15,16]. Even when patients achieve optimal glycemic control, early detection and treatment of hypertension are mandatory in the pediatric population with T1D to prevent micro- and macrovascular complications during adolescence and adulthood [17].

Studies underscore the importance of genetic influence on variations in blood pressure, giving the information that 20–40% of variations in blood pressure are genetically determined [18]. Many genes have been examined for their influence on the formation of diabetes complications. The most studied ones are part of the renin–angiotensin system (RAS), which regulates blood pressure and salt homeostasis and has a role in hypertension development.

The angiotensin-converting enzyme (*ACE*) gene is located on chromosome 17q23 and contains 25 introns and 26 exons [19]. *ACE* rs1799752 polymorphism has three genotypes: DD, II, and ID [20]. The two alleles (I/D) differ in size due to the insertion of 287 base pairs of a DNA sequence in intron 16 of the *ACE* gene.

The gene for angiotensin II receptor type 1 (*AGTR1*) is located on chromosome 3q22 [21]. *AGTR1* A1166C polymorphism refers to the change of adenine (A) to cytosine (C) at position 1166 from the start codon in the 3′ untranslated region of the gene [22]. AGTR1 polymorphism has three genotypes: AA, AC, and CC. The *ACE I/D* and *AGTR1 A/C* gene polymorphisms, as part of the renin–angiotensin system (RAS), have been associated with the risk of hypertension, diabetes, CVD, and nondiabetic renal disease [23,24,25,26]. The genetic variants of *ACE* and *AGTR1* can be justifiably considered candidate genes related to the pathogenesis of hypertension in diabetes.

This study aims to show the distribution of different RAS gene polymorphisms—*ACE I/D* gene polymorphism and *AGTR1* gene polymorphism—in adolescents with hypertension and T1D in the population of Serbia and analyze the association between *ACE* and *AGTR1* genotypes, blood pressure, and the diurnal variation in blood pressure (dipping status).

## 2. Materials and Methods

### 2.1. Patients

A cross-sectional study was conducted at the Endocrinology Department of the University Children’s Hospital in Belgrade, Serbia. The inclusion criteria required participants to be 11 years or older and to have a diagnosis of T1D lasting two or more years. The exclusion criteria included the use of renoprotective or antihypertensive therapy with ACE inhibitors, the presence of diabetic ketoacidosis at the time of inclusion, and impaired global renal function. We excluded patients undergoing treatment with ACE inhibitors because they were normotensive at the time of data collection for the study. This would have resulted in lower average blood pressure values for the hypertensive group, so the impact of gene polymorphisms on the development of hypertension might have been misinterpreted.

The study included 118 adolescents, 64 males and 54 females, with an average duration of diabetes of 5.5 ± 3.4 years. Participants were treated with multiple daily injections or continuous subcutaneous insulin infusion (CSII). The study protocol was approved by the Ethics Committee of the University Children’s Hospital in Belgrade (Resolution No. 14/84). Every participant and parent gave fully informed written consent to participate in the study.

This study is part of the same cohort as our previous investigation (doi: 10.1371/journal.pone.0312489) [27]. While the earlier research focused on genetic associations with microalbuminuria and diabetic nephropathy, the current study investigates hypertension, dipping status, and their relationships with *ACE* and *AGTR1* gene polymorphisms within the same cohort.

### 2.2. Analyses and Investigations

For each patient, we determined clinical parameters including age, sex, diabetes duration, body mass index (BMI), family history of CVD, and laboratory analyses including glycosylated hemoglobin A1c (HbA1c), total cholesterol, triglycerides, low-density lipoproteins (LDL), high-density lipoproteins (HDL), plasma creatinine, first void urine analysis, 24-h urine albumin excretion, and 24-h ambulatory blood pressure monitoring (24-h ABPM). Quantitative turbidimetric methods were used to detect glycosylated HbA1c and microalbuminuria (MA) levels. Both biomarkers were analyzed using the Siemens EXL 200 analyzer (Erlangen, Germany), while other blood tests were performed using standard laboratory measurements.

24-h ABPM measured blood pressure using an oscillometric method with the appropriate cuff size (on the non-dominant arm). The mean values of 24-h systolic blood pressure (sBP), diastolic blood pressure (dBP), mean arterial blood pressure (MAP), and diurnal variation in blood pressure were estimated. Hypertension was diagnosed when the mean 24-h sBP or dBP exceeded the 95th percentile for sex, age, and height. Non-dipping refers to a loss of mean daytime blood pressure that decreases less than 10% at night. Dipping is defined as a mean BP during the day decreased more than 10% during the night.

Patients with normal blood pressure were compared to those with hypertension, and patients with normal and altered nocturnal decline were also compared. The prevalence of *ACE I/D* and *AGTR1* genotypes was estimated in all groups.

### 2.3. Genetic Analysis

Deoxyribonucleic acid (DNA) was isolated from peripheral blood samples using a standardized protocol for DNA extraction with the commercially available QIAamp DNA Blood Mini Kit (Qiagen, Hilden, Germany). Subsequently, the Biospec-nano Shimadzu Spectrophotometer for Life Sciences (Shimadzu, Kyoto, Japan) was utilized to measure the concentrations. The regions containing the variants were first amplified using polymerase chain reaction (PCR), initiating the genotyping of the variants *ACE* rs1799752 and *AGTR1* rs5186. PCR amplifies DNA segments by binding them with specific oligonucleotides (primers), which are elongated by a polymerase, creating an additional strand of complementary sequences to the original base sequence. Consequently, part of the DNA segment in question was multiplied, making it easier to evaluate using various genotyping methods. The *ACE* rs1799752 variant was genotyped by assessing the sizes of the amplicons. Sanger sequencing, which is used for identifying single nucleotide changes and the presence of insertions or deletions of several base pairs in DNA segments with a maximum length of 800 to 1000 base pairs, was employed to detect the *AGTR1* rs5186 variant. The DNA segment was amplified, annealed to an oligonucleotide primer, and then extended by a DNA polymerase, incorporating both deoxynucleotide triphosphate molecules and chain-terminating dideoxynucleotide triphosphate molecules. The collected chromatographic data were analyzed using sequencing analysis software. The variants in the *ACE* and *AGTR1* genes were denoted based on the Ensembl database (https://www.ensembl.org/index.html, accessed on 26 February 2025), and the details relevant to this paper are provided below in Table 1.

### 2.4. Statistical Analysis

Data analysis involved both descriptive and analytical statistics. The Shapiro–Wilk test was applied to assess the normality of the distribution for continuous variables. Based on their distribution, continuous variables were expressed as mean ± standard deviation (SD) or median (minimum-maximum range). Categorical variables were summarized as counts and percentages. For categorical data analysis, either the Chi-squared test or Fisher’s exact test was used as appropriate. Continuous variables were compared between two groups using the independent samples *t*-test or the Mann–Whitney U test, depending on the normality distribution. Statistical significance was defined as *p* < 0.05. Univariate logistic regression analyses were conducted with hypertension and non-dipping as outcomes. Multivariable logistic regression with a backward Wald method was then used to select factors connected to hypertension and non-dipping. Variables strongly connected with these outcomes in univariate analysis (*p* < 0.1) were included in the multivariable model. Odds ratios (OR) with 95% confidence intervals (CI) were calculated, and the Hosmer–Lemeshow test was used to evaluate model fit. SPSS version 23.0 (SPSS Inc., Chicago, IL, USA) was used for statistical analysis.

## 3. Results

The clinical characteristics and laboratory analyses of patients are shown in Table 2. Normal blood pressure included 87% of our patients, while 13% of adolescents were diagnosed with hypertension. Patients with hypertension were predominantly female and demonstrated significantly poorer glycemic control, characterized by elevated HbA1c and triglyceride levels. Moreover, they had higher BMI values and required larger daily insulin doses (*p* < 0.05). To investigate the factors associated with the development of hypertension, both univariate and multivariate logistic regression analyses were conducted. The results of these analyses, summarized in Table 3, indicate that BMI (OR 1.32, 95% CI: 1.07–1.64), HbA1c (OR 2.24, 95% CI: 1.39–3.61), and insulin dose per kilogram (OR 26.43, 95% CI: 2.64–264.90) were significant predictors of hypertension in the multivariate analysis. There were no significant differences between the two groups in terms of age, duration of diabetes, family history of cardiovascular disease, or the number of patients experiencing nocturnal blood pressure decline. Additionally, no significant differences were found in HDL, LDL levels, proteinuria, or microalbuminuria between the groups.

The prevalence of *ACE* genotypes in the hypertensive group was 20% II, 66.7% ID, and 13.3% DD, which did not differ significantly from the group with normal blood pressure (29.1% II, 53.4% ID, and 17.5% DD) as shown in Table 4. Different *ACE* genotypes did not exhibit significant differences in blood pressure values, as indicated in Table 5.

The prevalence of AGTR1 genotypes in the hypertensive group was 40% AA, 53.3% AC, and 6.7% CC, which did not differ significantly from the group with normal BP (52.4% AA, 39.8% AC, and 7.8% CC), as shown in Table 6. The different *AGTR1* genotypes did not show significant differences in BP values, as detailed in Table 7. Additionally, there were no significant differences in genotype distribution between patients with a family history of CVD and hypertension and those without hypertension (*p* 0.285).

The non-dipping phenomenon was recognized in 45.8% of the study population. Non-dippers had a significantly higher MAP but a lower dBP, as shown in Table 2. No statistically significant differences were found in other parameters between the two groups regarding clinical characteristics and laboratory measurements, as indicated in Table 2. The prevalence of ACE and AGTR1 genotypes did not differ significantly between dippers and non-dippers, as shown in Table 4 and Table 6.

## 4. Discussion

This study aimed to detect hypertension and non-dipping phenomenon in adolescents with T1D in a population of Serbia and to determine its relationship with clinical data and genetic risk factors, including various variants of the *ACE* and *AGTR1* gene polymorphisms. Identifying risk factors for chronic diabetes complications is highly important, given the rising prevalence among youth. In our study group, poor glycemic control and female gender were identified as risk factors for hypertension. Similar findings were observed in other studies [28]. Adolescents with hypertension had higher BMI and triglyceride levels. Obesity and elevated BMI are known risk factors for hypertension [29,30]. Patients with hypertension required higher insulin doses, likely due to their increased body mass.

A single genetic polymorphism of a gene such as *ACE* or *AGTR1* may contribute to the risk of cardiovascular disease (CVD) and hypertension, either alone or in conjunction with other genetic factors. Environmental factors may modify the cumulative effects of common genetic polymorphisms. Moreover, genetic factors may affect the patient’s response to therapy. The unquestionable reason for the involvement of the RAS in the pathogenesis of hypertension is the effective treatment with *ACE* inhibitors and angiotensin receptor blockers (ARBs), which act through the inhibition of RAS. Furthermore, ARBs reduce proteinuria, making them especially helpful in children with diabetic nephropathy [31].

*ACE* and *AGTR1* gene polymorphisms are studied as risk factors for CVD and DN. Higher plasma *ACE* activity is associated with the development of DN, arterial hypertension, LVH, and myocardial infarction [32,33,34,35]. Elevated plasma and tissue *ACE* activity correlates with the D allele, while ID heterozygotes are linked to intermediate *ACE* levels [20]. Consequently, the DD genotype is most closely associated with hypertension and other chronic complications such as CVD and DN [36]. In the analysis by Staessen et al., left LVH, as a complication of prolonged hypertension, was associated with the *ACE I/D* polymorphism, but only in untreated adults [15]. In that study, patients with the DD genotype had a 192% increased risk of LVH compared to II homozygotes. Other studies have indicated a connection between the I allele and hypertension, insulin resistance, and metabolic syndrome [37,38,39]. Marre et al. showed that patients with the II genotype had a significantly lower risk of developing DN [40]. Our study found no association between *ACE* gene polymorphism and hypertension. Similar results are shown in other studies [41,42,43,44]. The ID genotype was more prevalent in patients with hypertension and non-dippers, but this was not statistically significant.

*AGTR1* seems to interpose the physiological effects of angiotensin II, which are crucial for regulating blood pressure, thus *AGTR1* polymorphism affects blood pressure control. *AGTR1* polymorphism has been connected with essential hypertension, LVH, myocardial infarction [45], and DN when associated with poor glycemic control [46]. The relationship between AGTR1 and hypertension combined with renal injury was shown before [47]. So far, the AA genotype has been associated with the development of DN, while the CC genotype has been shown to have the opposite effect [48,49]. Most of our hypertensive patients had the AC genotype of the *AGTR1* gene, while the AA genotype was the most frequent among patients with no dipping, although this finding lacked statistical significance.

The loss of nocturnal decline may be an additional factor contributing to the development of hypertension and the progression of target organ damage associated with diabetes. Genetic risk factors play a significant role in the loss of nocturnal blood pressure reduction, alongside clinical risk factors and autonomic neuropathy. In a study involving 238 adolescents and young adults with T1D, Deja G. et al. demonstrated that subjects exhibiting the non-dipping phenomenon were significantly older and had a longer duration of T1D [50]. Our patients with the non-dipping phenomenon exhibited higher MAP without any other differences in clinical data or laboratory analyses.

In addition to clinical and laboratory factors that have already been proven to be risk factors for hypertension, we found no association between genetic factors, such as ACE and AGTR1 gene polymorphisms, and the onset of hypertension or the non-dipping phenomenon in our patients. There were no differences in the distribution of *ACE I/D* or *AGTR1* genotypes among adolescents with and without hypertension or between dippers and non-dippers. According to the literature, we can conclude that the greatest importance of determining *ACE I/D* gene polymorphism lies in the management of chronic complications in patients with diabetes. In patients with the DD genotype, blood pressure should be monitored more frequently, as they are at a higher risk for hypertension and LVH.

One significant limitation of our study is the relatively low number of patients with hypertension, which may have impacted the power of our statistical analyses. Future studies with larger cohorts are needed to validate our results and provide a more comprehensive understanding of the potential genetic predisposition to hypertension in adolescents with type 1 diabetes.

## 5. Conclusions

Our results indicate that clinical factors, such as a higher body mass index, elevated triglycerides, suboptimal glycemic control, and female gender, are more predictive of hypertension development than genetic risk factors like *ACE* and *AGTR1* gene polymorphisms. Possible reasons for these results include the short diabetes duration, the young age of our patients, and the relatively small study group. Many patients are likely to develop hypertension in the future, which has to be taken into account. Future research will be necessary to verify these findings in larger sample studies and to investigate the genetic predisposition for hypertension development.

## Figures and Tables

**Table 1:** The details about analyzed ACE and AGTR1 polymorphism.

Gene	*ACE*	*AGTR1*
rs number	rs1799752	rs5186
Genome version	ENST00000290866.10	ENST00000349243.8
Genotype	(Insertion) c.2306-105_2306-104insTTTTTTTTTTTGAGACGGAGTCTCGCTCTGTCGCCCATACAGTCACTTTT (Deletion) c.2306-105_2306-104del	c.*86A>C
Chromosomal position	Chromosome 17:63488530-63488543 (forward strand)	Chromosome 3:148742201 (forward strand)

ACE—angiotensin-converting-enzyme, AGTR1—angiotensin II receptor type 1.

**Table 2:** Demography and clinical characteristics of the patients.

	Total (*n* = 118)	Hypertension Status			Dipping Status		
		HT (+) N 15 (13%)	HT (−) N 103 (87%)	*p*	Non-Dippers N 54 (46%)	Dippers N 64 (54%)	*p*
Female *n* (%)	54 (45.8)	11 (73.3)	43 (41.7)	0.022	23 (42.6)	31 (48.4)	0.525
Male *n* (%)	64 (54.2)	4 (26.7)	60 (58.3)		31 (57.4)	33 (51.6)	
Age (years) *	15.5 ± 2.3	15.5 ± 2.6	14.3 ± 2.3	0.077	14.3 ± 2.2	14.6 ± 2.5	0.568
Diabetes duration (years) *	5.5 ± 3.4	6.9 ± 3.3	5.3 ± 3.4	0.052	5.5 ± 3.5	5.4 ± 3.3	0.856
BMI (kg/m^2^) *	20.5 ± 3.0	22.0 ± 3.5	20.3 ± 2.9	0.022	20.0 ± 2.9	20.5 ± 3.2	0.368
HbA1c % †	7.9 (5.6–13.3)	9.0 (6.7–11.7)	7.7 (5.6–13.3)	0.001	8.1 (5.8–12.6)	7.8 (5.6–13.3)	0.136
Total cholesterol †	4.6 (2.0–7.9)	5.1 (3.0–7.9)	4.7 (2.0–7.7)	0.129	4.7 (3.3–7.9)	4.8 (2.0–7.7)	0.825
LDL cholesterol †	2.4 (0.5–6.2)	2.7 (1.7–6.2)	2.4 (0.5–5.1)	0.112	2.4 (0.5–5.1)	2.5 (1.1–6.2)	0.630
HDL cholesterol *	1.8 ± 0.4	1.6 ± 0.4	1.8 ± 0.4	0.130	1.8 ± 0.4	1.7 ± 0.4	0.591
Triglyceride †	0.7 (0.2–10.6)	1.1 (0.3–5.8)	0.7 (0.2–10.6)	0.040	0.8 (0.4–5.8)	0.7 (0.2–10.6)	0.638
Insulin dose/kg *	0.8 ± 0.3	1.0 ± 0.3	0.8 ± 0.3	0.005	0.8 ± 0.2	0.8 ± 0.3	0.456
CSII *n* (%)	25 (21.2)	4 (26.7)	21 (20.4)	0.532	13 (24.1)	12 (18.8)	0.201
MDI *n* (%)	93 (78.8)	11 (73.3)	82 (79.6)		41 (75.9)	52 (81.2)	
Microalbuminuria †	0.8 (0.05–10.5)	0.8 (0.4–10.5)	0.8 (0.05–8.2)	0.332	0.8 (0.05–10.5)	0.8 (0.1–8.5)	0.459
Family history of CVD, *n* (%)	25 (21.2)	3 (20.0)	22 (21.4)	0.904	12 (22.2)	13 (20.3)	0.800
Proteinuria † (24 h urine)	0.1 (0.01–1.2)	0.1 (0.03–0.3)	0.1 (0.01–1.2)	0.948	0.1 (0.01–1.2)	0.1 (0.02–0.4)	0.514
Creatinine clearance †	112.9 (35.0–236.1)	112.3 (35.0–139.9)	113.0 (39.0–236.1)	0.249	114.8 (41.7–217.9)	112.5 (35.0–236.1)	0.831
sBP (mmHg) †	113.0 (85.0–132.0)	123.0 (118.0–132.0)	112.0 (85.0–130.0)	0.001	112.0 (85.0–132.0)	115.0 (102.0–127.0)	0.053
dBP (mmHg) †	68.0 (51.0–85.0)	78.0 (61.0–85.0)	67.0 (51.0–80.0)	0.001	63.0 (51.0–80.0)	70.0 (56.0–85.0)	<0.001
MAP *	79.0 ± 6.2	80.1 ± 8.5	78.9 ± 5.8	0.398	80.4 ± 6.0	77.6 ± 6.1	0.012
Diabetic nephropathy *n* (%)	15 (12.7)	3 (20.0)	12 (11.7)	0.364	10 (18.5)	5 (7.8)	0.082
Dippers *n* (%)	64 (54.2)	10 (66.7)	54 (52.4)	0.301	NA	NA	NA
Non-dippers *n* (%)	54 (45.8)	5 (33.3)	49 (47.6)				

HT—hypertension, BMI—body mass index, CSII—continuous subcutaneous insulin infusion, MDI—multiple day injection, CV—cardiovascular diseases, sBP—systolic blood pressure, dBP—diastolic blood pressure, MAP—mean arterial pressure, NA—not applicable. *—variables are presented as mean ± SD, †—variables are presented as median (min–max). Patients whose mean BP during the day decreased by less than 10% at night were defined as non-dippers.

**Table 3:** Logistic regression analysis of predictors of hypertension.

	Univariate Logistic Regression		Multivariate Logistic Regression	
	OR	95% CI	OR	95% CI
Female sex	3.84	1.14–12.86		
BMI	1.22	1.02–1.45	1.32	1.07–1.64
HbA1c	1.89	1.28–2.80	2.24	1.39–3.61
Triglyceride	1.31	0.98–1.76		
Insulin dose/kg	14.47	2.01–104.38	26.43	2.64–264.90

BMI—body mass index, HbA1c—glycosylated HbA1c.

**Table 4:** Frequency of *ACE I/D* gene polymorphism in patients with hypertension or dipping phenomenon.

	Total N = 118	HT +	HT −	*p*-Value	Non-Dippers	Dippers	*p*-Value
DD	20 (16.9)	2 (13.3)	18 (17.5)	0.625	7 (13.0)	13 (20.3)	0.369
II	33 (28.0)	3 (20.0)	30 (29.1)		18 (33.3)	15 (23.4)	
ID	65 (55.1)	10 (66.7)	55 (53.4)		29 (53.7)	36 (56.3)	

Data are shown as *n* (%). HT—hypertension.

**Table 5:** Average blood pressure values in *ACE DD, DI, II* genotypes.

	DD	DI	II	*p*
sBP, mmHg Daytime	113.8 ± 9.8	112.9 ± 9.0	111.7 ± 6.3	>0.05
dBP, mmHg Daytime	67.6 ± 6.5	67.80 ± 6.8	67.4 ± 6.7	
Daytime MAP	78.8 ± 8.6	79.5 ± 6.0	77.6 ± 4.9	
sBP, mmHg Nighttime	105.5 ± 7.3	105.2 ± 8.93	105.2 ± 6.6	
dBP, mmHg Nighttime	62.7 ± 7.2	62.0 ± 7.4	61.3 ± 6.4	
Nighttime MAP	75.5 ± 8.1	74.6 ± 6.8	71.5 ± 11.5	
% fall sBP	11.1 ± 4.7	9.4 ± 4.7	8.8 ± 4.9	
% fall dBP	11.6 ± 5.9	11.5 ± 5.4	11.2 ± 5.6	
MAP	10.1 ± 5.0	9.9 ± 5.0	9.4 ± 4.9	

sBP—systolic blood pressure, dBP—diastolic blood pressure, MAP—median arterial pressure, % fall—a percentage of nocturnal decline. Variables are presented as mean ± SD.

**Table 6:** Frequency of *AGTR1 A/C* gene polymorphism in patients with hypertension or dipping phenomenon.

	Total N = 118	HT +	HT −	*p*-Value	Non-Dippers	Dippers	*p*-Value
AA	60 (50.8)	6 (40.0)	54 (52.4)	0.608	27 (50.0)	33 (51.6)	0.976
AC	49 (41.5)	8 (53.3)	41 (39.8)		23 (42.6)	26 (40.6)	
CC	9 (7.6)	1 (6.7)	8 (7.8)		4 (7.4)	5 (7.8)	

Data are shown as *n* (%). HT—hypertension.

**Table 7:** Average BP values in *AGTR1 AA, AC, CC* genotypes.

	AA	AC	CC	*p*
sBP, mmHg Daytime	112.2 ± 8.5	113.0 ± 8.3	114.9 ± 9.2	>0.05
dBP, mmHg Daytime	67.6 ± 6.8	68.0 ± 6.8	66.7 ± 6.4	
MAP Daytime	78.7 ± 5.9	79.0 ± 6.7	81.0 ± 5.8	
sBP, mmHg Nighttime	105.4 ± 8.0	105.3 ± 8.3	104.4 ± 6.9	
dBP, mmHg Nighttime	62.6 ± 6.8	61.4 ± 7.7	60.2 ± 5.0	
MAP Nighttime	73.1 ± 10.2	74.1 ± 6.6	78.1 ± 5.9	
% fall sBP	9.1 ± 5.0	10.2 ± 4.7	7.9 ± 3.9	
% fall dBP	10.4 ± 5.3	13.1 ± 5.4	9.4 ± 5.4	0.017
MAP	9.5 ± 5.0	10.5 ± 4.8	7.9 ± 5.3	>0.05

sBP—systolic blood pressure, dBP—diastolic blood pressure, MAP—median arterial pressure, % fall—percentage of nocturnal decline. Variables are presented as mean ± SD.

## Data Availability

Dataset available on request from the authors.
